# Commensal *Staphylococci* attenuate *Staphylococcus aureus* skin colonization and inflammation via AHR-dependent signaling

**DOI:** 10.3389/fimmu.2025.1726557

**Published:** 2025-12-01

**Authors:** Jule Riebelmann, Nicole Kienzle, Birgit Sauer, Birgit Schittek

**Affiliations:** Department of Dermatology, University Hospital Tuebingen, Tuebingen, Germany

**Keywords:** skin, skin microbiome, skin immunity, aryl hydrocarbon receptor, *Staphylococcus aureus*

## Abstract

**Introduction:**

*Staphylococcus aureus* is the leading cause of bacterial skin infections in several inflammatory skin diseases, however, is rarely detected on healthy skin. Skin barrier defects, such as in atopic dermatitis, promote *S. aureus* colonization by yet unknown mechanism. In our previous work we found that in healthy skin commensal staphylococci including Staphylococcus epidermidis and Staphylococcus lugdunensis (SL) protect against *S. aureus* skin colonization, however, the microbiome-mediated protection is lost in inflammatory skin. Here, we investigated how microbiome-derived factors contribute to skin defense under homeostatic and inflammatory conditions.

**Methods:**

We examined how bacterial conditioned media (BCM) from *S. epidermidis* and *S. lugdunensis* influence immune responses in primary human keratinocytes, human skin explants, and 3D skin reconstructs. Immune signaling was assessed using LEGENDplex cytokine profiling, RT2 Profiler PCR arrays, and western blotting. To investigate how BCM pretreatment limits *S. aureus* colonization, we performed inhibitor studies with a focus on aryl hydrocarbon receptor (AHR) signaling. The effects of BCM under inflammatory conditions were analyzed in tape-stripped human skin explants and 3D skin models with an atopic dermatitis–like phenotype.

**Results:**

We show that released factors from SE and SL reduce *S. aureus* skin colonization by inducing antimicrobial peptides (AMPs) and suppressing inflammatory responses in the skin. Both, factors released by SE and SL, limit *S. aureus*-induced immune activation in the skin by dampening inflammatory signaling, reducing reactive oxygen species, and suppressing expression of danger-associated molecular patterns (DAMPs). We show that this anti-inflammatory effect is mediated by activation of aryl hydrocarbon receptor (AHR) signaling in keratinocytes. Mechanistically, SE and SL membrane vesicles are involved in activating AHR signaling in keratinocytes via direct vesicle-cell contact as well as by bacterial tryptophan metabolites. This protective effect is lost in inflamed skin, where it instead exacerbated inflammation due to impaired AHR activity in inflamed skin. Interestingly, co-treatment of human AD-like skin equivalents with released SE factors together with an AHR ligand effectively reduces *S. aureus* colonization pointing out a potential novel AHR- and microbiome-based therapeutic strategy in AD.

**Discussion:**

Together, these findings highlight a context-dependent role of microbiome-derived factors in shaping cutaneous immunity and underscore the therapeutic potential of restoring AHR signaling to enhance the skin’s defense against *S. aureus*, particularly in inflammatory disorders such as AD.

## Graphical Abstract

**Figure:**
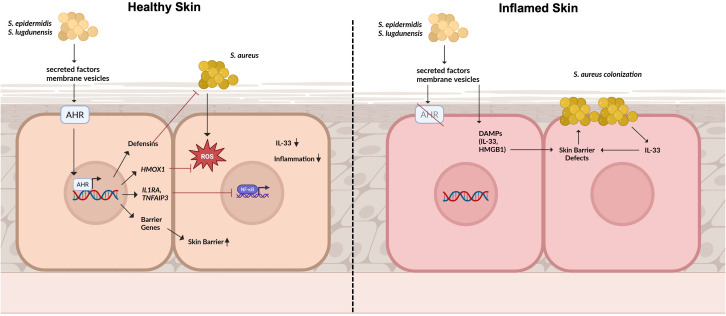
Role of aryl hydrocarbon receptor (AHR) signaling in maintaining skin homeostasis and modulating S. aureus colonization: In healthy skin, secreted factors and membrane vesicles derived from the skin microbiome activate AHR signaling in keratinocytes. This pathway induces the expression of antimicrobial peptides (AMPs), antioxidative genes such as HMOX1, and anti-inflammatory mediators that collectively prevent S. aureus-induced inflammation and colonization. AHR activation also promotes the expression of skin barrier genes, thereby reinforcing epidermal integrity. On the other hand, in inflamed skin, impaired AHR signaling diminishes these protective effects. Here, microbiome-derived factors can exacerbate inflammation by inducing damage-associated molecular pattens (DAMPs) which further compromise the skin barrier and favour S.aureus colonization.

## Introduction

*Staphylococcus aureus* (*S. aureus*) is a leading cause of skin and soft tissue infections that can progress to systemic, life-threatening disease (1). About 30% of humans are either transiently or permanently asymptomatically colonized by *S. aureus* predominantly on the nose and *S. aureus* colonization is a risk factor for subsequent infection (2, 3). *S. aureus* skin colonization is frequently detected in atopic dermatitis (3), where barrier defects and inflammation promote its colonization (4). Beyond passive colonization, *S. aureus* actively exacerbates inflammation and barrier disruption, establishing a self-reinforcing disease cycle (5–7). This is predominantly provided by the expression of a variety of virulence factors *S. aureus* expresses that help driving skin barrier defects and inflammation such as phenol-soluble modulins (PSMs), pore-forming toxins, superantigens as well as extracellular enzymes and cell wall associated proteins (1, 5, 8, 9). Moreover, its overrepresentation is associated with reduced microbial diversity, highlighting the role of the skin microbiome in regulating *S. aureus* colonization (10). Reduction of *S. aureus* colonization on the skin can limit inflammation and help prevent infections, particularly in conditions such as AD (11).

Skin commensals are integral components of the skin microbiome and actively create an environment that restricts *S. aureus* colonization (12–17). We and others previously demonstrated that the skin commensals *S. epidermidis* and *S. lugdunensis* inhibit *S. aureus* skin colonization partially by inducing host-derived antimicrobial responses (14–19). These host responses synergize with commensal-derived antimicrobial peptides (AMPs) to strengthen colonization resistance (14). Beyond their antimicrobial activity, skin commensals also contribute to the establishment and maintenance of a functional epidermal barrier (20–24). A key mechanism involves activation of aryl hydrocarbon receptor (AHR) signaling, which is engaged by skin commensals and plays an important role in supporting barrier integrity and antimicrobial defense (20, 25, 26). One potent group of AHR ligands is represented by tryptophan metabolites such as indoles, either derived endogenously or from skin commensals (27). Dysregulation of AHR signaling has been implicated in inflammatory skin diseases including AD and targeting AHR signaling has been proposed as a potential treatment option (26, 28–32).

We showed previously that the protective mechanism of the skin microbiome depends on skin barrier integrity. Upon skin barrier disruption, *S. epidermidis* fails to prevent *S. aureus* colonization (33). In the context of AD, it may even shift toward a proinflammatory role, aggravating skin inflammation (34–37). The mechanism underlying this dual behavior remains poorly understood. Elucidating the context-dependent effects of skin commensals is therefore critical to understand their contribution to host defense and to explore microbiome derived factors as therapeutic strategies against *S. aureus* in inflammatory skin diseases.

In this work, we aim to analyze how the skin microbiome shapes skin immune responses and protects against *S. aureus* skin colonization in healthy skin and how this identified mechanism can be used in a therapeutic setting to protect inflamed AD-skin against *S. aureus* colonization.

## Materials and methods

### Bacterial strains and culture conditions

The *Staphylococcus* strains used in this study included *S. aureus* USA300 LAC (SA), *S. epidermidis* 1457 (2), and *S. lugdunensis* IVK28 (SL). All strains were kindly provided by the working group of Andreas Peschel from the University of Tübingen. Bacterial cultures were grown aerobically in tryptic soy broth (TSB) at 37°C and 180 rpm. All experimental procedures employed bacteria in the logarithmic growth phase, corresponding to an optical density (9) of 0.5. To prepare *Staphylococcus* bacterial conditioned medium (BCM), 500 ul of an overnight culture was added to 50 mL of keratinocyte base medium (CELLnTEC (CnT), CnT-07). Following 18h of incubation at 37 °C and 180 rpm, an OD of 1 was set before the bacterial culture was centrifuged and subsequently filter sterilized.

### Cell culture

Primary human keratinocytes (PHKs) and primary human fibroblasts were isolated from human foreskin from donors up to 10 years after routine circumcision from the Loretto Clinic in Tübingen upon informed consent of patients as described elsewhere (14, 33). Briefly, subcutaneous fat and blood vessels were removed, and the foreskin was cut into small pieces. The tissue fragments were incubated overnight in epidermal keratinocyte medium with supplements containing 10 μg/ml gentamycin, 0.25μg/ml amphotericin B, and 10 mg/ml Dispase (9) to digest the basal lamina. The following day, the epidermis was separated from the dermis and incubated in 0.05% trypsin-EDTA (Merck Millipore) for 30 min. Digestion was stopped by adding RPMI medium (ThermoFisher Scientific) supplemented with 10% FCS (FBS, Biochrom). Single-cell suspensions were obtained by filtration through a 100 μm cell strainer (Corning Incorporated). After centrifugation, cells were resuspended in epidermal keratinocyte medium with supplements. Keratinocyte and fibroblast isolation from human foreskin was approved by the ethics committee of the medical faculty of the University Tübingen (654/2014BO2) and performed according to the principles of the Declaration of Helsinki.

PHKs were cultured in collagen-coated flasks in keratinocyte base medium (CellnTec, CnT-07) until confluency. 24h prior to the experiments, differentiation was induced in PHKs by changing the medium to CnT supplemented with 1.7 mM CaCl2. PHKs were treated with BCM, membrane vesicles (MVs) or AHR ligands for the indicated time points. For AHR inhibitor treatment, PHKs were treated with CH-223191 2h before BCM stimulation.

### Adhesion & invasion assay

Eighteen hours after treatment of PHKs with BCM, AHR ligands or AHR inhibitor, the supernatant of the PHKs was removed, the cells washed twice with HBSS, and fresh CnT+CaCl2 medium was added to the cells. Afterwards, PHKs were infected with logarithmically growing *S. aureus* USA300 (MOI = 30) for 1.5h at 37°C. After infection, the supernatant was removed, PHKs were washed twice with HBSS and subsequently lysed in 250 µl lysis buffer (1x PBS + 10% Triton-X + 2.5% Trypsin). Serial dilutions of the lysate were plated onto TSB agar plates and incubated overnight at 37°C. The next day, CFUs were analyzed.

### 3D human skin equivalents

3D human skin equivalents (HSEs) were prepared as described previously (38). Briefly, primary human fibroblasts were seeded onto collagen matrices and cultured in FF medium for five days. Subsequently, primary keratinocytes were introduced into the wells, and the cultures were subjected to air-liquid interface conditions on day 12. To generate AD-like models, HSEs were treated with Th2 cytokines (20 ng/ml IL-4, 10 ng/ml IL-13, 10ng/ml IL-5) for three days at the end of the HSE culture as described previously (38). Afterwards, HSEs were treated with BCM SE, FICZ, or a combination of both. After 18h, HSEs were lysed for RNA isolation, and supernatants were collected for subsequent LEGENDplex analysis. For infection experiments, *S. aureus* USA300 (1x10^8 CFU) was topically applied to the epidermis of the HSE using 8 mm filter paper discs (Smart Practice) and incubated for 18h. To assess bacterial colonization, the HSEs were homogenized with scissors, and serial dilutions of the homogenate were plated on TSB agar plates and incubated overnight at 37°C. CFUs were analyzed the next day.

### Human skin explants

The subcutaneous fat and blood vessels of human foreskin was removed, and skin explants were incubated in CnT supplemented with gentamycin and amphotericin for 3h. For induction of skin barrier defects, skin explants were tape-stripped by repeated stripping of adhesive tape (fixomull). Six mm of skin punches were transferred into transwell inserts which were placed above 300 µl CnT medium. BCM SE was topically applied for 18h.

### RNA isolation, cDNA synthesis and quantitative reverse transcription-polymerase chain reaction

RNA from PHKs, human skin explants or human skin equivalents was isolated with the NucleoSpin RNA Kit from Macherey-Nagel according to the manufacturer’s protocol. For cDNA generation, 2 µg of RNA was used, and cDNA synthesis was performed as described previously (14). QRT-PCR was performed in 10 µl reaction volume with SYBRGreen PCR Master Mix according to the manufacturer’s instructions using a LightCycler 96 (Roche Life Science). The primer sequences and respective annealing temperatures are listed in **Supplementary Table 1**.

### RT^2^-profiler™ array

PHKs or skin explants were treated with BCM for 18h before subsequent RNA isolation and cDNA synthesis. The antibacterial response RT Profiler Array from Qiagen (PAHS-148Z) was used according to the manufacturer’s instructions. Genes included in the RT Profiler Array are listed in **Supplementary Table 2**.

### Western blot

Western Blot was used to analyze signaling pathways activated by treatment in PHKs using whole-cell lysates. Cells were lysed in a buffer supplemented with both protease and phosphatase inhibitors to preserve protein integrity. The resulting lysates were subjected to SDS-PAGE and transferred onto PVDF membranes. Membranes were blocked for one hour in PBS containing 0.1% Tween-20 and 5% dry milk and subsequently incubated overnight at 4°C with primary antibodies against p65, phosphorylated p65, ERK, phosphorylated ERK, and β-actin (each diluted 1:1000; Cell Signaling). Following three washes in PBS with 0.1% Tween-20, membranes were incubated in a horseradish peroxidase-conjugated anti-rabbit secondary antibody (1:2000; Cell Signaling). Protein bands were visualized using ECL detection reagents (ThermoFisher Scientific) and imaged with an Amersham Imager 600 (GE Healthcare).

### LEGENDplex™ multiplex cytokine analysis

To assess cytokine production, 25 µl of supernatants were collected from PHKs. The analysis was conducted using the LEGENDplex™ Human Inflammation Panel 1 (Biolegend), following the protocol provided by the manufacturer. Samples were processed on a BD LSRII flow cytometer and subsequently analyzed using LEGENDplex™ software (Biolegend).

### Enzyme-linked immunosorbent assay (ELISA)

Levels of cytokines in cell culture supernatants were quantified using DuoSet ELISA Kits (R&D Systems) according to the manufacturer’s protocol. 96-well ELISA plates (Nunc) were first coated with 50 µl of capture antibody and incubated overnight at 4°C. The following day, plates were washed three times with PBS containing 0.05% Tween-20, then blocked with PBS supplemented with 1% BSA for one hour at room temperature (3). After another round of three washes, 50 µl of either cell culture supernatant or standard was added to each well, followed by a 2-hour incubation at RT. Plates were again washed and then treated with a biotin-labeled detection antibody for 2 hours at RT. This was followed by a 20-minute incubation in the dark with HRP-streptavidin. After a final set of washes, TMB substrate (Cell Signaling Technology) was added to initiate color development. The reaction wash stopped with 2N sulfuric acid, and absorbance was read at 450 nm.

### HMGB1 ELISA

ELISA plates (Nunc) were coated with 50 µl of cell culture supernatant or recombinant HMGB1 standards (starting from 8 ng/ml) overnight at 4°C. After three washing steps with PBS containing 0.05% Tween-20, 50 µl of an anti-HMGB1 antibody (1:120; R&D Systems) were added to each well and incubated for 2h at RT. Subsequently, the plate was washed three times and subsequently 50 µl of an HRP-conjugated detection antibody (anti-mouse IgG; 1:3000) was added to the wells and incubated for 2h at RT. After another three washing steps, 50 µl of TMB substrate was added to the wells to initiate color development. After 20 minutes, a stopping solution (2N sulfuric acid) was added to the wells and absorbance was read at 450 nm.

### Isolation of membrane vesicles

Membrane vesicles were isolated following a previously established protocol (39, 40). Briefly, culture supernatants from Staphylococci, harvested after 6 hours of growth (late exponential phase), were first sterile-filtered and then loaded onto 100 kDa molecular weight cutoff centrifugal concentrator cartridges (Vivaspin 20; Sartorius). Samples were centrifuged at 3000 x g for 20 minutes, and the retained >100 kDa fraction was collected and resuspended in 1 mL of TSB. This suspension was then mixed with ExoQuick reagent (System Biosciences) at a 5:1 ratio and incubated overnight at 4°C. The following day, membrane vesicles were pelleted by centrifugation at 1500 x g for 30 minutes and subsequently resuspended in 1 ml of fresh PBS. Protein concentration of MVs was analyzed by BCA assay. For disintegration, MVs were incubated in an ultrasonic bath (Allpax palssonic, UD06, 40 kHz) for 30 min. To deplete MVs from the BCM, the BCM was ultrafiltrated with 100 kDa centrifugal concentrator cartridges (Vivaspin 20; Sartorius) for 30 min at 3000xg. The <100 kDa fraction represents the MV depleted BCM.

### Viability assay

To assess the impact of the different treatments on the viability of PHKs, a 4-methylumbelliferyl heptanoate (MUH)-based assay was used. Following treatment, PHKs were incubated with MUH at a final concentration of 100 ug/ml in PBS for 1 hour at 37°C. Fluorescence was then measured with excitation at 355 nm and emission at 460 nm to determine viability based on enzymatic activity.

### Quantification and statistical analysis

Statistical analyses were conducted using GraphPad Prism 10 (GraphPad Software, Inc.). Differences between samples were evaluated using either unpaired, two-tailed Student’s *t*-tests or one-way analysis of variance (ANOVA) followed by Dunnett’s multiple comparisons tests, as specified in the respective figure legends. All data visualizations were created with GraphPad Prism 10.

## Results

### Commensal-released factors activate skin host defense pathways to protect against *Staphylococcus aureus* skin colonization

**Figure 1:**
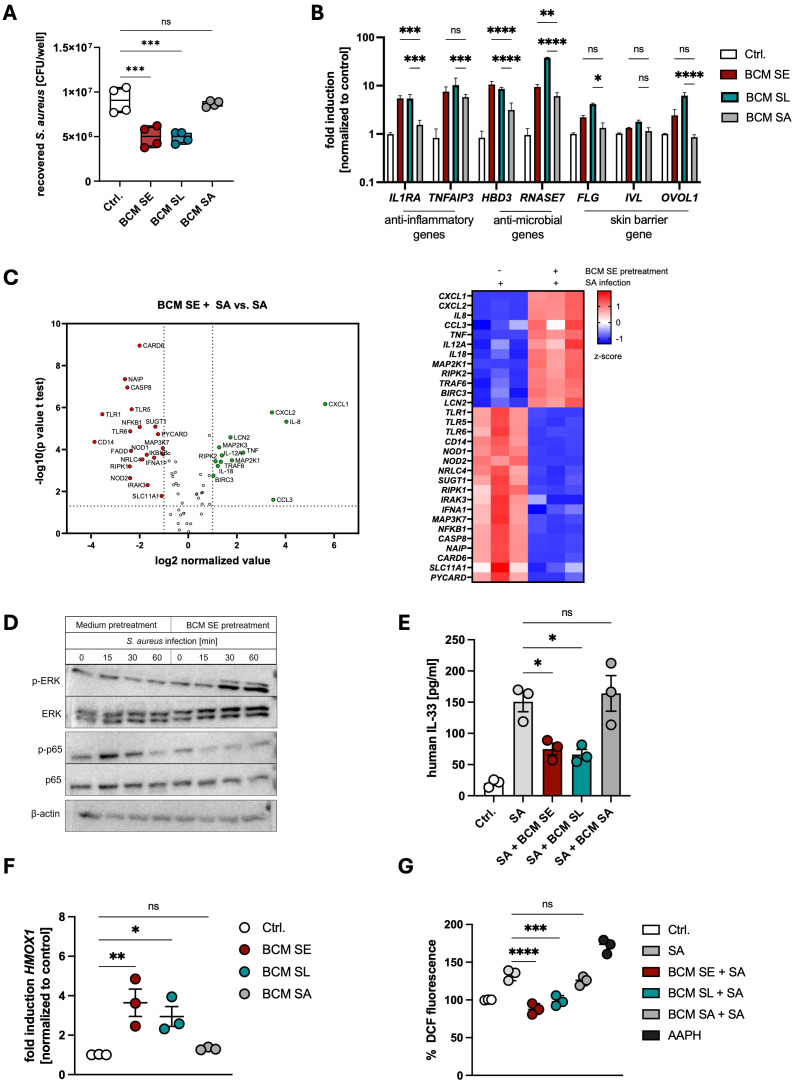
Commensal factors activate host defense pathways to protect against *Staphylococcus aureus* skin colonization. **(A)** PHKs were treated for 18h with BCM of SE, SL or SA before subsequently being infected with *S. aureus* (MOI = 30). After 1.5h, PHKs were lysed and CFU were analyzed. Shown is the mean of four biological experiments with one dot representing one biological replicate +/- SEM. Significant differences to the control were analyzed by one-way ANOVA. **(B)** Gene expression analysis in PHKs after 18h treatment with BCM SE, SL, SA. Shown is the mean of three biological replicates +/- SEM. Statistical differences to the BCM SA sample were analyzed by 2-way ANOVA. **(C)** RT-Profiler Array of PHKs after 1.5h *S. aureus* infection +/- pretreatment with BCM SE (18h). Shown is the mean of three biological replicates. Statistical differences in gene expression between the samples were analyzed by unpaired t-tests. **(D)** Signaling pathway activation in PHKs after different infection times with *S. aureus* with and without BCM SE pretreatment. Shown is one representative experiment of three independent experiments **(E)** IL33 ELISA of supernatants of PHKs after *S. aureus* infection +/- BCM SE, SL, SA pretreatment (18h); Ctrl. = uninfected PHKs. Shown is the mean of three biological replicated +/- SEM. One dot represents one biological replicate. Statistical differences to SA sample were analyzed by one-way ANOVA. **(F)** Gene expression of *HMOX1* in PHKs after 18h treatment with BCM SE, SL, SA; Ctrl. = untreated PHKs. Shown is the mean of three biological replicates +/- SEM with one dot representing one biological experiment. Statistical differences to the control were analyzed by one-way ANOVA. **(G)** Measurement of reactive oxygen species in PHKs after *S. aureus* infection +/- pretreatment with BCM SE, SL or SA; Ctrl. = uninfected, untreated PHKs; AAPH = positive control. Shown is the mean of three biological replicates with one dot representing one biological replicate. Statistical differences to the SA sample were analyzed by one-way ANOVA. Statistical significance = *p < 0.05; **p < 0.01; ***p < 0.001; ****p < 0.0001. PHKs, primary human keratinocytes; BCM, bacteria conditioned medium; SE, *S. epidermidis*; SL, *S. lugdunensis*; SA, *S. aureus*; MOI, multiplicity of infection; CFU, colony forming units.

To unravel the mechanism of the protective effect of skin commensals on *S. aureus* skin colonization, we treated PHKs with BCM of SE or SL before *S. aureus* infection. Subsequently, we performed an adhesion and invasion assay to determine CFUs. We found that pretreatment of PHKs with BCM of both skin commensals reduced *S. aureus* adherence on PHKs, while BCM of the pathogen SA did not (**Figure 1A**), confirming our previous studies (14, 33, 39). Using an RT-PCR Profiler Array we determined the antibacterial response induced in PHKs by BCM treatment. Interestingly, treatment of PHKs with BCM SE or SL increased expression of several proinflammatory cytokines such as *IL1B*, *IL8*, *TNF* and *IL18* and genes involved in NOD-like (e. g. *RIPK2*), inflammasome-related (e. g. *NLRP3*), MAPK (e. g. *MAPK3*) and NFκB (*NFKBIA*) signaling, whereas genes involved in type I interferon signaling (e. g. *IFNA1*), stress-related pathways (e. g. *JUN*) and apoptosis (e. g. *FADD*) are downregulated (**Supplementary Figures S1A, B**). In contrast, treatment of PHKs with BCM of SA increased mainly expression of proinflammatory cytokines and chemokines such as *IL8*, *IL6*, *CXCL1* and *CXCL2* and genes involved in apoptosis (e. g. *FADD*), whereas expression of genes involved in TLR (e. g. *TLR1*)- and NOD-like (e. g. *RIPK1*) signaling are decreased (**Supplementary Figure S1C**).

We extended the analysis by measuring expression of genes involved inflammation, antimicrobial response and skin barrier by qRT-PCR, Legendplex analysis and ELISA. We found that BCM of both commensals significantly induced the expression of the anti-inflammatory genes *IL1RA* and *TNFAIP3* as well as genes encoding the antimicrobial peptides (AMPs) HBD3 and RNase7 and in most cases to a higher extent than BCM SA (**Figure 1B**). A similar kind of induction was seen for skin barrier related genes encoding filaggrin (*FLG*), involucrin (*IVL*) and OVO-like protein 1 (*OVOL1*) (**Figure 1B**). The results suggest that released factors of the skin commensals SE and SL induce a protective environment mainly by inducing expression of anti-inflammatory and antimicrobial genes as well as skin barrier genes in PHKs. These genes were also induced by released factors of SA, however, they induced in general a more pro-inflammatory phenotype with the secretion of much higher levels of pro-inflammatory cytokines.

Next, we analyzed how pretreatment of PHKs with BCM of skin commensals shapes the immune response towards *S. aureus* infection. For this, we treated PHKs for 18h with BCM of SE or as a control only with medium before infecting PHKs with *S. aureus* (MOI = 30) for 1.5 h and then analyzed gene expression via an RT-PCR Profiler Array. Interestingly, upon BCM SE pretreatment, genes involved in NOD-like- (e. g. *NOD1*, *2*), TLR- (e. g. *1, 6, MYD88*), NFκB (e. g. *NFkB1, IKBKB1*) and apoptosis (e. g. *CASP8, FADD*) signaling were significantly downregulated in PHKs compared to *S. aureus* infection alone, whereas genes involved in MAPK signaling (e. g. *MAP2K1, MAP2K3*) are upregulated (**Figure 1C**). Furthermore, chemokines known to attract monocytes and neutrophils to sites of infections such as *CXCL1*, *CXCL2*, *CCL3* and *IL8* were upregulated in PHKs by BCM SE pretreatment compared to *S. aureus* infection alone (**Figure 1C**).

The data suggest that pretreatment of PHKs with BCM of SE reduces NFκB signaling and activates MAPK signaling. Indeed, we confirmed by western blot analysis that BCM of SE increased phosphorylation of ERK in a time-dependent fashion and reduced the increased phosphorylation of p65 seen after *S. aureus* infection (**Figure 1D**, **Supplementary Figure S1E**). This change in signaling pathway activity was not detected upon priming the PHKs with BCM SA (**Supplementary Figure S1F**). Interestingly, pretreatment of PHKs with the BCM of the skin commensals SE and SL, but not with BCM of SA, reduced the secretion of IL-33, an alarmin and danger-associated molecular pattern (DAMP) involved in skin barrier damage (**Figure 1E**). Furthermore, pretreatment of PHKs with BCM of the skin commensals SE and SL, but not of SA, induced the expression of the antioxidant defense gene *HMOX1* and reduced the induction of oxidative stress in PHKs after *S. aureus* infection (**Figures 1F, G**).

Together, these results indicate that factors secreted by the skin commensals SE and SL markedly enhance the expression of anti-inflammatory, antimicrobial, and barrier-associated genes, thereby initiating innate immune alert pathways that prime the skin for pathogen exposure while attenuating inflammatory and oxidative responses induced by *S. aureus* infection.

### Activation of AHR signaling mediates commensal-mediated protection against *S. aureus*-induced skin inflammation

**Figure 2:**
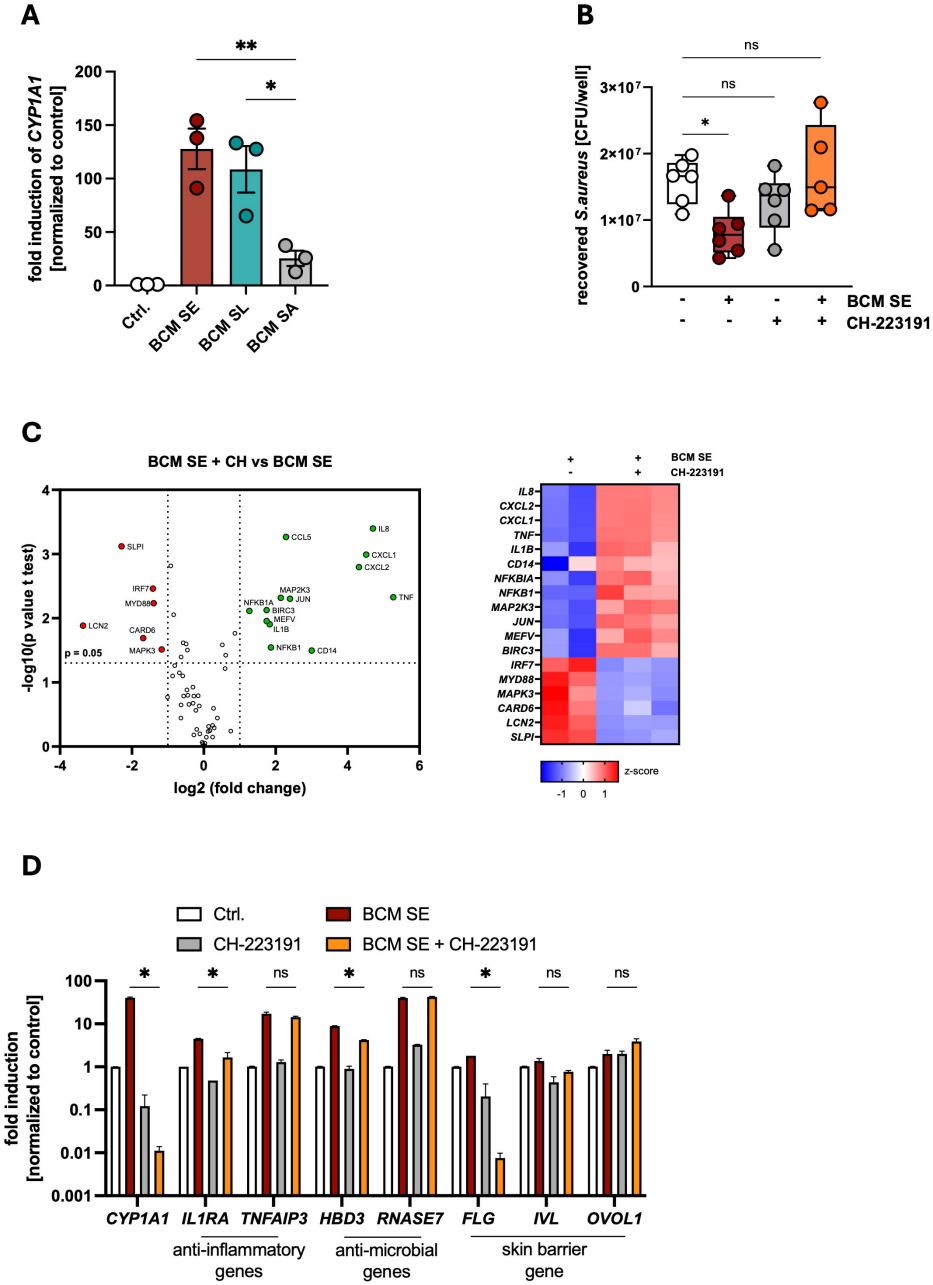
Activation of AHR signaling mediates commensal-dependent protection against *S. aureus*-induced skin inflammation. **(A)** Gene expression of *CYP1A1* in PHKs after 4h treatment with BCM SE, SL or SA. Ctrl. = untreated PHKs. Shown is the mean of three biological replicates +/- SEM with one dot representing one biological replicate. Statistical differences to the BCM SA sample were analyzed by one-way ANOVA. **(B)** PHKs were treated with BCM SE (18h) or left untreated in the presence or absence of AHR inhibitor CH-223191 before subsequent infection with *S. aureus* (MOI = 30) for 1.5h. PHKs were lysed and CFUs were analyzed. Shown is the mean of six biological experiments +/- SEM with one dot representing one biological replicate. Statistical differences to the control were analyzed by one-way ANOVA. **(C)** RT-Profiler Array of PHKs after 18h treatment with BCM SE +/- CH-223191. Data represent the mean of two to three biological replicates. Statistical differences in gene expression between the samples were analyzed by unpaired t-tests. **(D)** Gene expression in PHKs after 18h BCM SE treatment +/- CH-223191. Shown is the mean of three biological replicates +/- SEM. Statistical differences between samples were analyzed by multiple unpaired t-tests. Statistical significance = *p < 0.05; **p < 0.01; ***p < 0.001; ****p < 0.0001. PHKs, primary human keratinocytes; BCM, bacteria conditioned medium; SE, *S. epidermidis*; SL, *S. lugdunensis*; SA, S*. aureus*; AHR, aryl hydrocarbon receptor; SD, standard deviation; SEM, standard error of the mean; MOI, multiplicity of infection; CFU, colony forming units.

The aryl hydrocarbon (AHR) is a critical regulator of skin barrier function and antimicrobial response towards pathogens (20, 41). Indeed, we found a strong induction of the AHR-target gene *CYP1A1* in PHKs 3h after stimulation with BCM SE and SL, while BCM SA induced *CYP1A1* expression at lower levels (**Figure 2A**). To investigate the role of AHR signaling in the protective effect of skin commensals, we treated PHKs with the AHR-inhibitor CH-223191 which significantly inhibited activation of AHR signaling in PHKs by BCM SE and BCM SL (**Supplementary Figure S2A**). Interestingly, inhibition of AHR signaling abolished the protective effect of BCM SE against *S. aureus* skin colonization (**Figure 2B**). This indicates that AHR signaling is involved in the protective effect of skin commensals towards *S. aureus* skin colonization.

To analyze the mechanisms of the AHR-mediated protective effect we first performed a RT-PCR Profiler Array of PHKs pretreated with BCM SE with and without the AHR inhibitor CH-223191. Interestingly, in the absence of AHR signaling, BCM SE induces a more pro-inflammatory phenotype in PHKs including genes involved in neutrophil recruitment (e.g. *IL8*), NFκB- and MAPK- signaling pathways (e.g. *NFKB1*; *MAP2K3*) (**Figure 2C**). CH-223191 treatment also significantly reduced the BCM SE and SL-mediated induction of anti-inflammatory and antimicrobial genes as well as skin barrier genes (**Figure 2D**, **Supplementary Figure S2B**). In addition, AHR inhibition significantly reduces the secretion of the proinflammatory cytokines IL-1α and IL-36α induced by BCM SE and SL, whereas IL-8 secretion is not affected (**Supplementary Figures S2C, D**). Interestingly, in the absence of AHR signaling, BCM SE and SL significantly induce gene expression and protein secretion of DAMPs like IL-33 (**Supplementary Figures S2E, F**) and HMGB1 (**Supplementary Figures S2G, H**) from PHKs.

In summary, the data indicate that BCM of skin commensals induce a protective environment by activation of AHR signaling, which reduces skin inflammation and increases skin barrier function leading to reduced *S. aureus* skin colonization. The data suggest that in environments with defective AHR signaling, BCM of skin commensals leads to an exacerbation of skin inflammation.

### Treatment of AD-skin with released factors of skin commensals together with activators of AHR signaling restores the protective effect against *S. aureus* colonization

**Figure 3:**
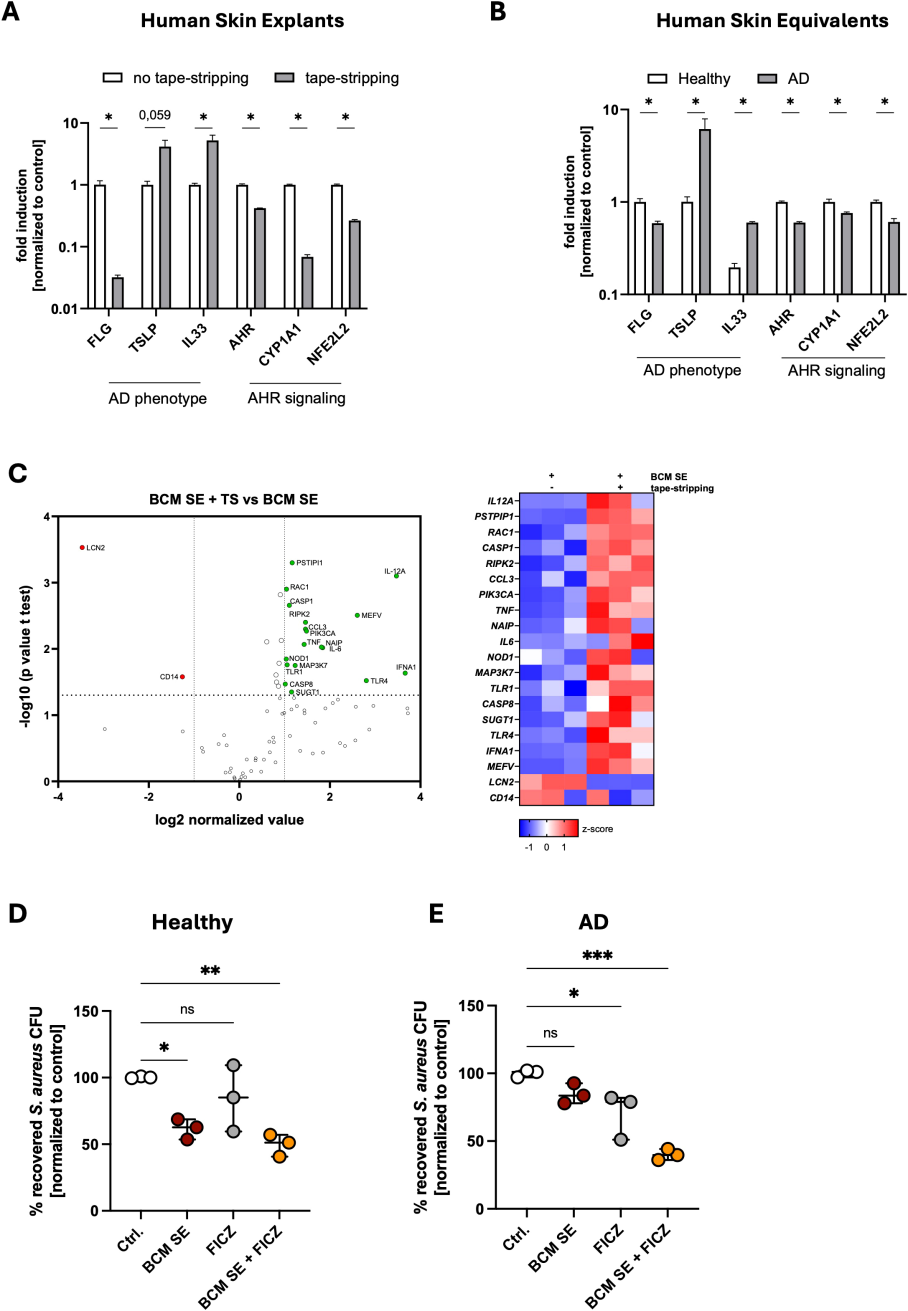
Released factors of skin commensals enhance skin inflammation in an atopic dermatitis environment. **(A, B)** Gene expression in human skin explants either untreated of 24h after tape-stripping (TS) **(A)** or 3D skin reconstructs with a healthy or AD phenotype **(B)**. Shown is the mean of three biological replicates +/- SEM. One biological replicate represents one individual donor. Statistical differences in gene expression between the samples were analyzed by multiple unpaired t-tests. **(C)** RT-Profiler Array of skin explants treated for 18h with BCM SE +/- TS. Shown is the mean of three biological replicates with one biological replicate representing one individual donor. Statistical differences in gene expression between the samples were analyzed by unpaired t-tests. **(D, E)** 3D human skin explants with a healthy **(D)** or AD **(E)** phenotype were treated with 18h with BCM SE, FICZ or the combination of both before epicutaneous application of *S. aureus* (1x10^8) for 18h. Shown is the mean of three biological replicates +/- SEM with one dot representing one biological replicate. Statistical differences to the control were analyzed by one-way ANOVA. Ctrl. = untreated reconstructs. Statistical significance = *p < 0.05; **p < 0.01; ***p < 0.001; ****p < 0.0001. TS, tape-stripping; AD, atopic dermatitis; SEM, standard error of the mean; BCM, bacteria conditioned medium; SE, *S. epidermidis*.

Previous results of our group showed that the protective effect of BCM SE depends on skin barrier integrity (33). In a tape-stripping model, which resembles the skin barrier defects of atopic dermatitis (1), *S. aureus* colonization is enhanced and cannot be reduced by pretreatment with BCM of SE (33). To study the mechanism of the missing protective effect in an inflammatory environment, we used two human 3D AD models. First, we induced a skin barrier defect in human skin explants by repeated tape-stripping, and second, we used an established 3D human skin equivalent where an AD phenotype was induced by treatment with the Th2 cytokines IL-4 and IL-13 (38). The AD phenotype was confirmed in both models by reduced expression of *FLG*, encoding for filaggrin, and enhanced expression of *TSLP* and *IL33*, both inflammatory markers for AD (**Figures 3A, B**). Interestingly, in both models, genes associated with AHR signaling (*AHR*, *CYP1A1*, *NEF2L2*), were downregulated, suggesting impaired AHR signaling (**Figures 3A, B**). We then tested how BCM SE affects the immune response in an AD environment. For this, we applied BCM SE on tape-stripped or non-tape-stripped human skin explants for 18h and analyzed immune responses using an RT-PCR Profiler array. We found that several pro-inflammatory genes are significantly upregulated by BCM SE in tape-stripped skin compared to intact skin (**Figure 3C**). The data suggests that BCM of skin commensals exacerbates skin inflammation in AD skin and shifts the response towards skin commensals from an anti-inflammatory and antimicrobial to an enhanced pro-inflammatory response.

Since several genes involved in AHR signaling are expressed at reduced levels in the AD models (see **Figures 3A, B**), we hypothesized that AHR signaling might be defective in the AD models resulting in exacerbation of skin inflammation after contact with skin commensals. Therefore, to test whether activation of AHR signaling in the AD models restore the protective effect mediated by skin commensals, we topically applied for 18h BCM SE, the AHR ligand FICZ, or the combination of both to 3D skin models with either a healthy or an AD phenotype before we topically applied *S. aureus* to the models for 18h and subsequently analyzed CFUs (**Figures 3D, E**). In the healthy model, BCM SE treatment significantly reduced *S. aureus* colonization and the combination of BCM SE and FICZ did not provide a significant additive effect (**Figure 3D**). In the AD model, the BCM SE alone did not significantly reduced *S. aureus* colonization. Although the FICZ treatment reduced *S. aureus* colonization in the AD model, the combination of both FICZ and BCM SE provided a strong significant reduction in recovered *S. aureus* from the skin (**Figure 3E**). These data indicate that treatment of AD skin by a combination of released factors of skin commensals together with activators of AHR signaling restores the protective effect of BCM of skin commensals in an AD skin environment.

### Membrane vesicles of skin commensals are involved in AHR activation in PHKs

Finally, we analyzed which released factors mediate the protective effect of skin commensals. We first tested representative bacterial tryptophan metabolites indole-3-propionate (I3P) and indole-3-aldehyde (I3A), known to be present in the conditioned medium of SE and SL (42), and for comparison the host-derived tryptophan metabolite L-kynurenine (L-K). Interestingly, the bacterial metabolites I3P and I3A induced strong AHR signaling in PHKs, whereas the host-derived ligand L-K showed only weak activity (**Supplementary Figures S3A–C**). Furthermore, consistent with the dose-dependent AHR induction, I3A treatment significantly reduced *S. aureus* colonization in a dose-dependent manner while neither I3P nor L-K provided a protective effect (**Supplementary Figures S3D–F**). It might be that the ligand for AHR activation is derived from PHKs after BCM stimulation.

**Figure 4:**
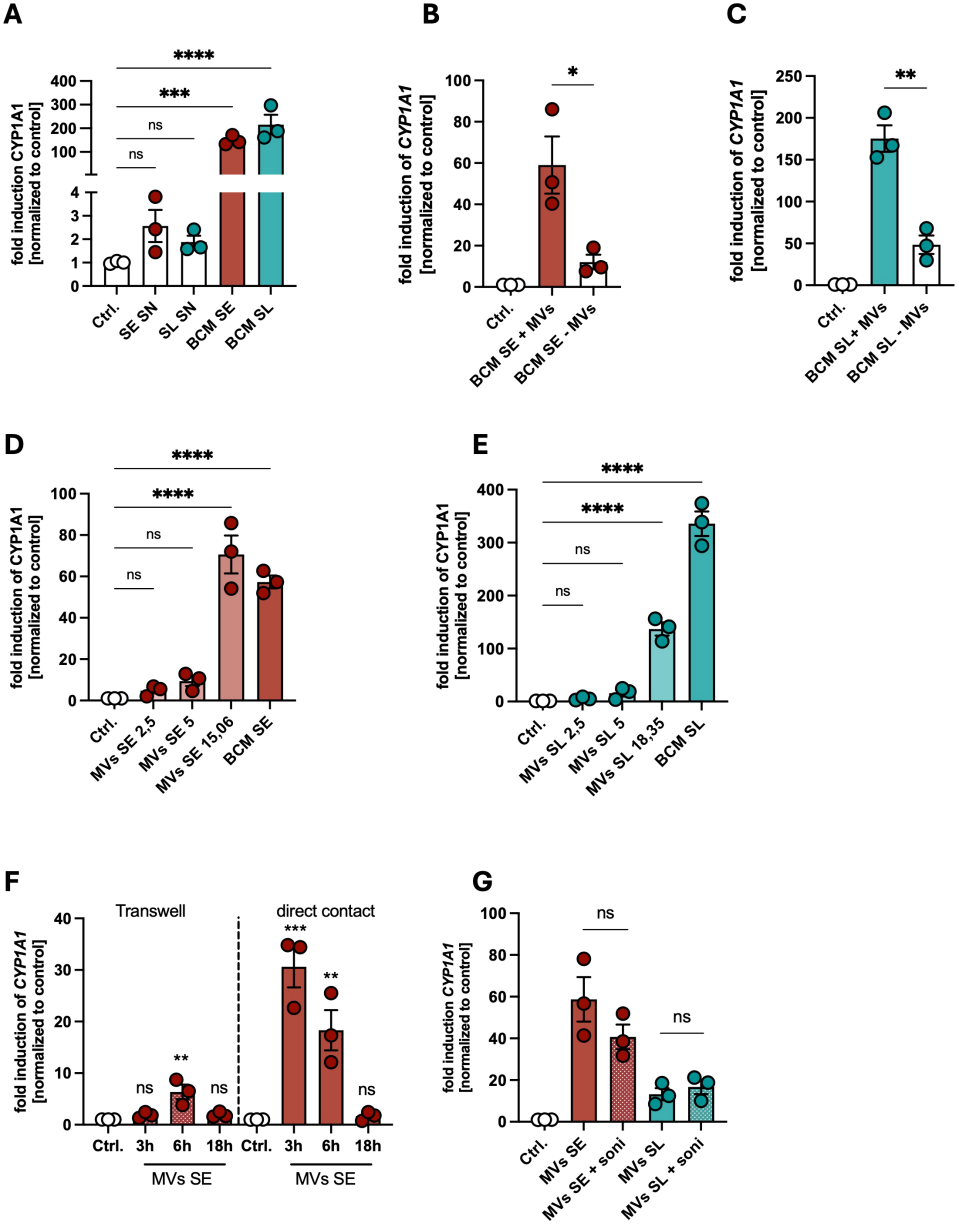
Membrane vesicles of skin commensals are involved in AHR activation in PHKs. **(A)** Gene expression of *CYP1A1* in PHKs treated with SE SN, SL SN, BCM SE or BCM SL for 18h; Ctrl. = untreated PHKs. Shown are the means of three biological replicates with one dot representing one biological replicate. Statistical differences in gene expression to the control were analyzed by one-way ANOVA. **(B, C)** Gene expression of *CYP1A1* in PHKs treated with BCM SE **(B)** or BCM SL **(C)** +/- MVs for 4h. Ctrl. = untreated PHKs. Shown is the mean of three biological replicates +/- SEM with one dot representing one biological replicate. Statistical differences between the samples were analyzed by unpaired t-tests. **(D, E)** Gene expression of *CYP1A1* in PHKs treated with BCM SE **(D)** or BCM SL **(E)** or different concentrations of the respective MVs. Ctrl. = untreated PHKs. Shown is the mean of three biological replicates with one dot representing one biological replicate. Statistical differences in gene expression to the control were analyzed by one-way ANOVA. **(F)** Gene expression of *CYP1A1* in PHKs treated for different time points with MVs of SE either in direct contact or separated by a transwell insert. Shown is the mean of three biological replicates +/- SEM with one dot representing one biological replicate. Statistical differences to the control were analyzed by one-way ANOVA. **(G)** Gene expression of *CYP1A1* in PHKs treated with MVs of SE or SL with and without prior disruption by ultrasonication. Ctrl. = untreated PHKs. Shown are the means of three biological replicates +/- SEM with one dot representing one biological replicate. Statistical differences in gene expression between the samples were analyzed by unpaired t-tests. Statistical significance = *p < 0.05; **p < 0.01; ***p < 0.001; ****p < 0.0001. SE, *S. epidermidis*; SL, *S. lugdunensis*; SN, supernatant; MVs, membrane vesicles; BCM, bacteria conditioned medium.

To test whether BCM of SE and SL induce the release of AHR ligands in PHKs which subsequently activate AHR signaling in PHKs, we treated PHKs with BCM of SE or SL for 4h, removed the BCM, washed the PHKs twice with HBSS and added fresh medium to the PHKs. After 18h incubation time, we collected the supernatant, filter-sterilized it and added it to new PHKs for 4h and subsequently analyzed CYP1A1 expression in those PHKs. We found that compared to treatment with BCM of SE or SL they induced only weakly AHR signaling in fresh PHKs, resembling the effect of the host-derived tryptophan metabolite L-K (**Figure 4A**). All these data indicate that metabolites from the skin commensals activate AHR signaling in PHKs.

Bacterial membrane vesicles (MVs) are part of the released factors found in BCM. Our previous data showed that skin commensals can release MVs which can mediate a protective against *S. aureus* skin colonization (39). To test whether MVs are able to activate AHR signaling in PHKs, we depleted MVs from BCM of the skin commensals as described previously (17), treated PHKs with BCM SE or SL with or without MVs and analyzed induction of *CYP1A1* in PHKs. Interestingly, AHR signaling in PHKs was significantly reduced when MVs were removed from the BCM of skin commensals (**Figures 4B, C**). Vice versa, we found that purified MVs of SE or SL induce *CYP1A1* expression in a dose-dependent fashion (**Figures 4D, E**). The highest concentration of MVs used resembles the MV content in the respective BCM. Interestingly, we found that MVs of SE are the main part in the BCM involved in activation of AHR signaling in PHKs in contrast to those of SL, where the BCM is nearly 4-fold more able to induce *CYP1A1* expression compared to the respective MVs concentration found in the BCM (**Figure 4E**). In contrast to MVs of the skin commensals, MVs of SA did not induce AHR signaling in PHKs (**Supplementary Figures S3G, H**). This indicates that there exist some strain-specific differences in the potential of MVs of the respective skin commensal to activate AHR signaling.

Do MVs have to be in direct contact with PHKs to activate AHR signaling? We tested that in a transwell system, where MVs of SE are either in direct contact with PHKs or are separated from PHKs by a transwell insert. MVs in direct contact with PHKs induced *CYP1A1* expression in a time-dependent fashion, whereas *CYP1A1* induction was much weaker after indirect contact (**Figure 4F**). Furthermore, it seems that integrity of the MVs is not required for AHR activation, as sonication, which successfully disrupted MVs (**Supplementary Figure S3I**), did not impair their ability to induce AHR signaling in PHKs (**Figure 4G**).

Together, these data indicate that MVs of skin commensals are able to activate AHR signaling by direct contact in PHKs, however there exist strain-specific differences in the efficiency of MVs in AHR activation. Furthermore, the bacterial metabolite I3A induced strong AHR signaling in PHKs and is able to reduce *S. aureus* adherence to PHKs.

## Discussion

*S. aureus* is a leading cause of skin infections and is frequently detected on AD skin, where barrier defects and inflammation promote its colonization and worsen disease (4, 43). In healthy skin, commensal bacteria limit *S. aureus* colonization (13–15, 19, 44, 45). However, this protective effect is dependent on skin integrity, and skin barrier disruption can impair commensal control of *S. aureus* (34, 36, 37, 46). In this work, we aimed to understand how the skin microbiome regulates *S. aureus* colonization in both healthy and barrier-disrupted and inflamed skin.

Interestingly, we detected that BCM SE, BCM SL and BCM SA treatment induced similar genes in PHKs in our RT Profiler Array. However, on protein level, only BCM SA induced a strong proinflammatory response involving the secretion of high levels of cytokines and chemokines whereas BCM SL and BCM SE only induced the secretion of a few selected cytokines. Therefore, we hypothesize that upon BCM SL and BCM SE treatment, the PHKs enter an alarmed state in preparation for pathogen encounters.

Consistent with the findings of others, we found that skin commensals induce the expression of anti-microbial and anti-inflammatory genes in PHKs (14, 25, 44). In line with the anti-inflammatory gene expression, BCM pretreatment also reduced skin inflammation induced by *S. aureus*. This indicates that the BCMs activate the antimicrobial defense of the skin while at the same time limit skin inflammation induced by pathogens. One way the skin commensals attenuate *S. aureus*-induced skin inflammation is by reducing the secretion of the DAMP IL-33. This is particularly relevant given IL-33’s central role in AD, where it activates type 2 innate lymphoid cells (ILC2s), drives Th2-skewed responses, regulates mast cell function, and impairs skin barrier integrity by downregulating epidermal differentiation markers such as filaggrin (47–51). A recent study showed that topical application of propionate, a microbial metabolite abundant on healthy skin but reduced in AD skin, diminished inflammation in a MC903-induced AD mouse model by inhibiting IL-33 production in keratinocytes (26). Consistently, treatment with the anti-IL-33 antibody etokimab in adults with moderate-to-severe AD reduces disease severity and limits inflammation by inhibiting neutrophil recruitment (52). In line with these observations, we previously demonstrated that, along with reducing *S. aureus* skin colonization, priming the skin with *S. epidermidis* decreased neutrophil infiltration following *S. aureus* colonization and mitigated *S. aureus*-induced inflammation (53). Collectively, these findings suggest that skin commensals limit *S. aureus*-induced inflammation by suppressing IL-33, and that this protective axis may be disrupted in AD due to microbiome dysbiosis and *S. aureus* overgrowth.

We found that the protective effects of the skin microbiome, including suppression of IL-33-mediated inflammation, enhancement of skin barrier function and reduction of *S. aureus* skin colonization, depend on activation of AHR signaling in keratinocytes. AHR signaling is increasingly recognized as a key pathway through which the skin microbiome maintains skin barrier integrity and limits inflammation, and it has emerged as a promising therapeutic target in AD. Dual AHR/Nrf2 activation reverses Th2 cytokine-mediated suppression of epidermal barrier genes following *S. aureus* colonization with a defined consortium restores barrier competence in an AHR-dependent manner (20, 45). Consistent with our findings, *S. epidermidis* activates AHR signaling in keratinocytes to induce innate immune pathway, including AMP expression (25). We extend these observations by showing that *S. lugdunensis* also activates keratinocyte AHR, a mechanism not previously described, and that MVs of skin commensals are potent AHR activators, with activity reduced upon MV depletion from conditioned medium.

Microbial tryptophan metabolites, particularly indole derivatives, represent potent AHR ligands and have been shown to enhance epithelial barrier function *in vitro* and *in vivo*. For example, colonization of gnotobiotic mice with a consortium of human skin commensals enriches indole-related metabolites compared to germ-free skin, improving barrier repair and function in AD-like models, while topical phytic acid increases tryptophan-metabolizing bacteria such as *S. epidermidis*, upregulates indole derivatives and activates AHR to promote barrier formation and ameliorate AD (27, 42) (54). In our study, I3A, a metabolite present in the supernatant of skin commensals (42), dose-dependently induced AHR signaling in keratinocytes and reduced *S. aureus* skin colonization.

Together, our findings identify AHR signaling as a central pathway by which skin commensals, via MVs and tryptophan-derived metabolites such as indole-3-aldehyde, enhance barrier function, suppress IL-33-driven inflammation and limit *S. aureus* colonization, a protective axis that appears diminished in AD (see **Graphical Abstract**).

Although *S. epidermidis* is generally considered a protective commensal of human skin, it fails to confer protection against *S. aureus* under conditions of barrier disruption (33). High-dose application of commensal skin bacteria to barrier impaired skin significantly delays barrier repair and amplifies cutaneous inflammation (46). Consistent with these observations, we found that exposure of tape-stripped human skin explants, which mimic AD-like barrier defects, to *S. epidermidis* conditioned medium markedly exacerbated skin inflammation. Multiple studies have demonstrated that in the context of AD, *S. epidermidis* can potentiate inflammatory responses. Its extracellular serine protease (Esp) activates IL-33 and promotes Th2 immune responses (36). Furthermore, *S. epidermidis* strains isolated from atopic lesional skin altered the morphology of 3D skin reconstructs, whereas strains from healthy skin did not. Healthy-skin isolates activated AHR/OVOL1 pathway and produced high levels of indole metabolites, especially indole-3-aldehyde, in co-culture with keratinocytes. In contrast, AD-associated strains failed to activate AHR/OVOL1 signaling, generated minimal indole metabolites, and disrupted key differentiation markers such as filaggrin (34). Consistently, indole-3-aldehyde levels are significantly lower in both lesional and non-lesional skin of AD patients compared to healthy individuals (55). This indicates that the capacity of *S. epidermidis* to induce AHR signaling is attenuate in AD. We further observed that in absence of AHR signaling in keratinocytes, *S. epidermidis*-derived factors elicited a markedly enhanced pro-inflammatory phenotype characterized by increased expression and secretion of DAMPs IL-33 and HMGB1. This suggests that impaired AHR signaling may amplify *S. epidermidis*-mediated inflammation, thereby contributing to AD pathogenesis.

AHR signaling itself appears dysregulated in AD. For instance, expression of the AHR target gene *CYP1A1* is reduced in lesional skin relative to healthy control (29). Similarly, we observed significant downregulation of AHR-related genes in two human AD-like models, 3D skin reconstructs treated with Th2 cytokines and tape-stripped human skin explants. We found that treatment of the AD-like 3D reconstructs with either BCM SE or an AHR-ligand FICZ alone did not provide protection against *S. aureus* colonization, however, the combination of BCM SE and FICZ restored the protective effect. This highlights the critical role of AHR activation in modulating *S. epidermidis* responses under barrier impairment and suggests a commensal-AHR combinatorial approach for AD therapy.

## Data Availability

The original contributions presented in the study are included in the article/**Supplementary Material**. Further inquiries can be directed to the corresponding author.
