# Supplementary material for: Commensal Staphylococci attenuate Staphylococcus aureus skin colonization and inflammation via AHR-dependent signaling
Source: Front Immunol. 2025 Dec 1;16:1726557. doi: 10.3389/fimmu.2025.1726557 (PMC12703969; doi:10.3389/fimmu.2025.1726557)
Supplement: Supplementary file 1 [file DataSheet1.pdf]

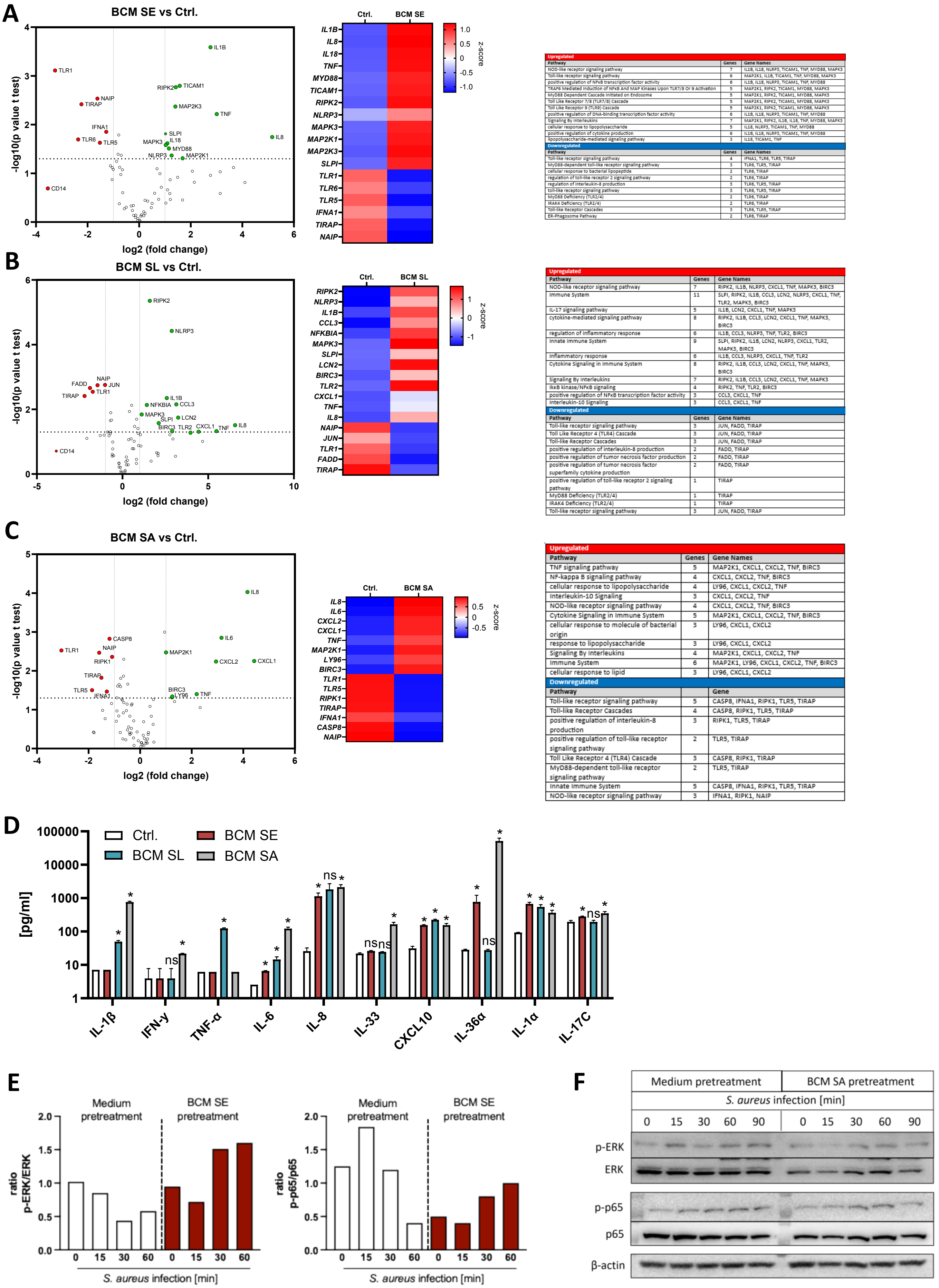

Upregulated		
**Pathway**	**Genes**	**Gene Names**
NOD-like receptor signaling pathway	7	IL1B, IL18, NLRP3, TICAM1, TNF, MYD88, MAPK3
Toll-like receptor signaling pathway	6	MAP2K1, IL1B, TICAM1, TNF, MYD88, MAPK3
positive regulation of NFκB transcription factor activity	6	IL1B, IL18, NLRP3, TICAM1, TNF, MYD88
TRAF6 Mediated Induction of NFκB And MAP Kinases Upon TLR7/8 Or 9 Activation	5	MAP2K1, RIPK2, TICAM1, MYD88, MAPK3
MyD88 Dependent Cascade Initiated on Endosome	5	MAP2K1, RIPK2, TICAM1, MYD88, MAPK3
Toll Like Receptor 7/8 (TLR7/8) Cascade	5	MAP2K1, RIPK2, TICAM1, MYD88, MAPK3
Toll Like Receptor 9 (TLR9) Cascade	5	MAP2K1, RIPK2, TICAM1, MYD88, MAPK3
positive regulation of DNA-binding transcription factor activity	6	IL1B, IL18, NLRP3, TICAM1, TNF, MYD88
Signaling By Interleukins	7	MAP2K1, RIPK2, IL1B, IL18, TNF, MYD88, MAPK3
cellular response to lipopolysaccharide	5	IL18, NLRP3, TICAM1, TNF, MYD88
positive regulation of cytokine production	6	IL1B, IL18, NLRP3, TICAM1, TNF, MYD88
lipopolysaccharide-mediated signaling pathway	3	IL18, TICAM1, TNF
**Downregulated**		
**Pathway**	**Genes**	**Gene Names**
Toll-like receptor signaling pathway	4	IFNA1, TLR6, TLR5, TIRAP
MyD88-dependent toll-like receptor signaling pathway	3	TLR6, TLR5, TIRAP
cellular response to bacterial lipopeptide	2	TLR6, TIRAP
regulation of toll-like receptor 2 signaling pathway	2	TLR6, TIRAP
regulation of interleukin-8 production	3	TLR6, TLR5, TIRAP
toll-like receptor signaling pathway	3	TLR6, TLR5, TIRAP
MyD88 Deficiency (TLR2/4)	2	TLR6, TIRAP
IRAK4 Deficiency (TLR2/4)	2	TLR6, TIRAP
Toll-like Receptor Cascades	3	TLR6, TLR5, TIRAP
ER-Phagosome Pathway	2	TLR6, TIRAP

Upregulated		
**Pathway**	**Genes**	**Gene Names**
NOD-like receptor signaling pathway	7	RIPK2, IL1B, NLRP3, CXCL1, TNF, MAPK3, BIRC3
Immune System	11	SLPI, RIPK2, IL1B, CCL3, LCN2, NLRP3, CXCL1, TNF, TLR2, MAPK3, BIRC3
IL-17 signaling pathway	5	IL1B, LCN2, CXCL1, TNF, MAPK3
cytokine-mediated signaling pathway	8	RIPK2, IL1B, CCL3, LCN2, CXCL1, TNF, MAPK3, BIRC3
regulation of inflammatory response	6	IL1B, CCL3, NLRP3, TNF, TLR2, BIRC3
Innate Immune System	9	SLPI, RIPK2, IL1B, LCN2, NLRP3, CXCL1, TLR2, MAPK3, BIRC3
Inflammatory response	6	IL1B, CCL3, NLRP3, CXCL1, TNF, TLR2
Cytokine Signaling in Immune System	8	RIPK2, IL1B, CCL3, LCN2, CXCL1, TNF, MAPK3, BIRC3
Signaling By Interleukins	7	RIPK2, IL1B, CCL3, LCN2, CXCL1, TNF, MAPK3
IkκB kinase/NFκB signaling	4	RIPK2, TNF, TLR2, BIRC3
positive regulation of NFκB transcription factor activity	3	CCL3, CXCL1, TNF
Interleukin-10 Signaling	3	CCL3, CXCL1, TNF
**Downregulated**		
**Pathway**	**Genes**	**Gene Names**
Toll-like receptor signaling pathway	3	JUN, FADD, TIRAP
Toll Like Receptor 4 (TLR4) Cascade	3	JUN, FADD, TIRAP
Toll-like Receptor Cascades	3	JUN, FADD, TIRAP
positive regulation of interleukin-8 production	2	FADD, TIRAP
positive regulation of tumor necrosis factor production	2	FADD, TIRAP
positive regulation of tumor necrosis factor superfamily cytokine production	2	FADD, TIRAP
positive regulation of toll-like receptor 2 signaling pathway	1	TIRAP
MyD88 Deficiency (TLR2/4)	1	TIRAP
IRAK4 Deficiency (TLR2/4)	1	TIRAP
Toll-like receptor signaling pathway	3	JUN, FADD, TIRAP

Upregulated		
**Pathway**	**Genes**	**Gene Names**
TNF signaling pathway	5	MAP2K1, CXCL1, CXCL2, TNF, BIRC3
NF-kappa B signaling pathway	4	CXCL1, CXCL2, TNF, BIRC3
cellular response to lipopolysaccharide	4	LY96, CXCL1, CXCL2, TNF
Interleukin-10 Signaling	3	CXCL1, CXCL2, TNF
NOD-like receptor signaling pathway	4	CXCL1, CXCL2, TNF, BIRC3
Cytokine Signaling in Immune System	5	MAP2K1, CXCL1, CXCL2, TNF, BIRC3
cellular response to molecule of bacterial origin	3	LY96, CXCL1, CXCL2
response to lipopolysaccharide	3	LY96, CXCL1, CXCL2
Signaling By Interleukins	4	MAP2K1, CXCL1, CXCL2, TNF
Immune System	6	MAP2K1, LY96, CXCL1, CXCL2, TNF, BIRC3
cellular response to lipid	3	LY96, CXCL1, CXCL2
**Downregulated**		
**Pathway**		**Gene**
Toll-like receptor signaling pathway	5	CASP8, IFNA1, RIPK1, TLR5, TIRAP
Toll-like Receptor Cascades	4	CASP8, RIPK1, TLR5, TIRAP
positive regulation of interleukin-8 production	3	RIPK1, TLR5, TIRAP
positive regulation of toll-like receptor signaling pathway	2	TLR5, TIRAP
Toll Like Receptor 4 (TLR4) Cascade	3	CASP8, RIPK1, TIRAP
MyD88-dependent toll-like receptor signaling pathway	2	TLR5, TIRAP
Innate Immune System	5	CASP8, IFNA1, RIPK1, TLR5, TIRAP
NOD-like receptor signaling pathway	3	IFNA1, RIPK1, NAIP

Figure S1: A-C: RT Profiler Array of PHKs after 18h treatment with BCM SE (**A**), BCM SL (**B**) or BCM SA (**C**). Shown is the mean of three biological replicates. Ctrl. = untreated PHKs. Statistical differences in gene expression to the control were analyzed by t-tests. **D:** Legendplex analysis of supernatants of PHKs after 18h treatment with BCM SE, SL or SA. Ctrl. = untreated PHKs. Shown is one representative experiment of three independent experiments. Statistical differences to the control were analyzed by one-way ANOVA. **E:** Quantification of the ratio of p-ERK/ERK and p-65/p65 protein expression in PHKs after different S. aureus infection times with and without previous BCM SE treatment. **F:** Western Blot of PHKs treated with either medium of BCM SA for 18h before S. aureus infection for different time points. Activation of p-ERK and p-p65 was analyzed. Actin was included as loading control. Statistical significance = (*$p<0.05$; **$p<0.01$; ***$p<0.001$; ****$p<0.0001$). PHKs = primary human keratinocytes; SE = *S. epidermidis*; SL = *S. lugdunensis*; SA = *S. aureus*; BCM = bacteria conditioned medium

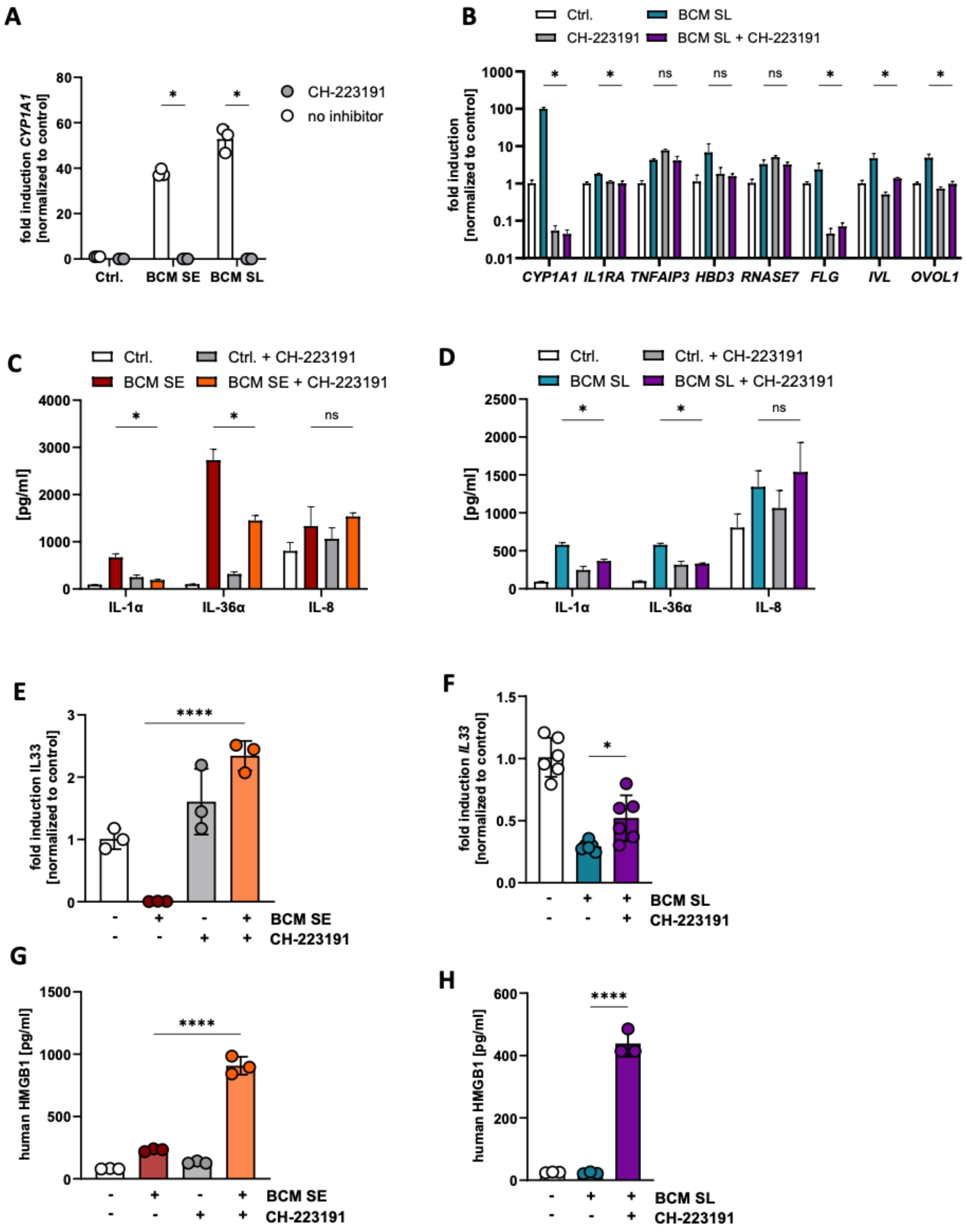

A
fold induction CYP1A1 [normalized to control]
80
60
40
20
0
Ctrl.
BCM SE
BCM SL
*
*
CH-223191
no inhibitor
B
Ctrl.
CH-223191
BCM SL
BCM SL + CH-223191
fold induction [normalized to control]
1000
100
10
1
0.1
0.01
* * ns ns ns * * *
CYP1A1 IL1RA TNFAIP3 HBD3 RNASE7 FLG IVL OVOL1
C
Ctrl.
BCM SE
Ctrl. + CH-223191
BCM SE + CH-223191
[pg/ml]
4000
3000
2000
1000
0
* * ns
IL-1α IL-36α IL-8
D
Ctrl.
BCM SL
Ctrl. + CH-223191
BCM SL + CH-223191
[pg/ml]
2500
2000
1500
1000
500
0
* * ns
IL-1α IL-36α IL-8
E
fold induction IL33 [normalized to control]
3
2
1
0
****
- + - + BCM SE
- - + + CH-223191
F
fold induction IL33 [normalized to control]
1.5
1.0
0.5
0.0
*
- + + BCM SL
- - + CH-223191
G
human HMGB1 [pg/ml]
1500
1000
500
0
****
- + - + BCM SE
- - + + CH-223191
H
human HMGB1 [pg/ml]
600
400
200
0
****
- + + BCM SL
- - + CH-223191

Figure S2: A: Gene expression of CYP1A1 in PHKs after 4h BCM SE/SL treatment +/- CH-223191. Shown is one representative experiment of three independent experiments +/- SD. Statistical differences were analyzed by multiple unpaired t-tests. **B:** Gene expression in PHKs after 18h treatment with BM SL +/- CH-223191. Shown is one representative experiment of three independent experiments +/- SD. Statistical differences were analyzed by multiple unpaired t-tests, **C&D:** ELISA of supernatants of PHKs treated with BCM SE (**C**) or BCM SL (**D**) for 18h +/- CH-223191. Shown is one representative experiment of three independent experiments. Statistical differences were analyzed by unpaired t-tests. **E&F:** Gene expression of IL-33 in PHKs after 18h treatment of BCM SE (**E**) or BCM SL (**F**) +/- CH-223191. Shown is one representative experiment of three independent experiments. Statistical differences were analyzed by unpaired t-tests. **G&H:** ELISA for HMGB1 in PHKs fater 18h treatment with BCM SE (**G**) or BCM SL (**H**) +/- CH-223191. Shown is one representative experiment of three independent experiments +/- SD. Statistical differences were analyzed by unpaired t-tests. Statistical significance = *p<0.05; **p<0.01; ***p<0.001; p<0.0001).PHKs = primary human keratinocytes; SE = S. epidermidis; SL = S. lugdunensis

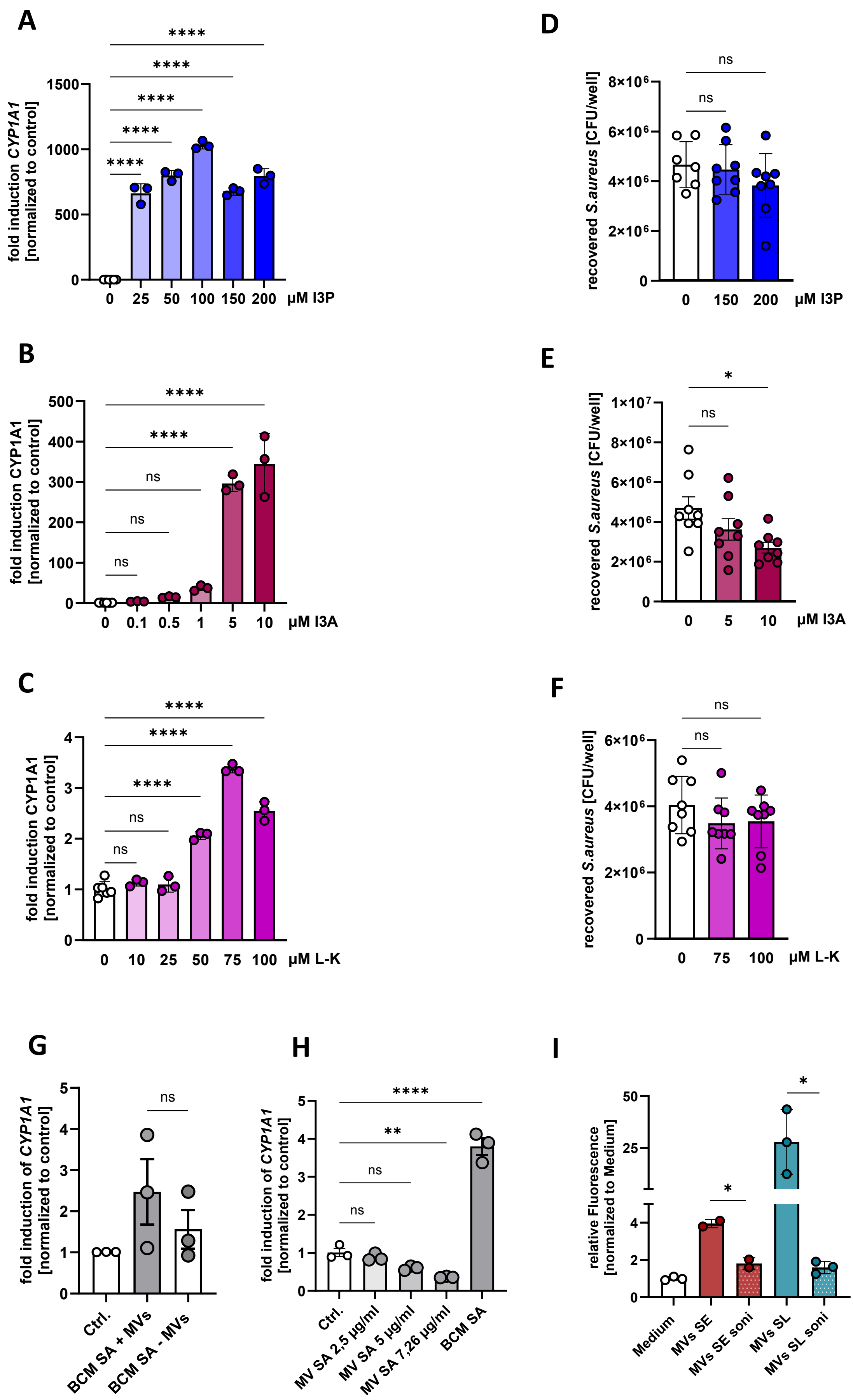
A
fold induction CYP1A1 [normalized to control]
μM I3P
0 25 50 100 150 200
****
ns
B
fold induction CYP1A1 [normalized to control]
μM I3A
0 0.1 0.5 1 5 10
C
μM L-K
0 10 25 50 75 100
D
recovered S.aureus [CFU/well]
0 150 200
E
0 5 10
*
F
0 75 100
G
fold induction of CYP1A1 [normalized to control]
Ctrl.
BCM SA + MVs
BCM SA - MVs
H
MV SA 2,5 µg/ml
MV SA 5 µg/ml
MV SA 7,26 µg/ml
BCM SA
**
I
relative Fluorescence [normalized to Medium]
Medium
MVs SE
MVs SE soni
MVs SL
MVs SL soni

Figure S3: A-C: Gene expression of CYP1A1 in PHKs treated with different concentrations of AHR ligands I3P (**A**), I3A (**B**) and L-K (**C**) for 4h. Shown is one representative experiment of three independent experiments +/- SD. Ctrl. = untreated PHKs. **D-F:** PHKs were treated with I3P (**D**); I3A (**E**), and L-K (**F**) for 18h before S. aureus infection (MOI = 30) for 1.5h. Subsequently, PHKs were lysed and CFU were analyzed. Shown is one representative experiment of two independent experiments +/- SD. Ctrl. = untreated PHKs. **G&H:** Gene expression of CYP1A1 in PHKs treated with BCM SA +/- MVs (**G**) or different concentrations of isolated MVs SA (**H**) for 4h. Shown is the mean of three biological replicates +/- SEM. **I:** FM646 fluorescence of MVs of SE and SL with and without ultrasonication disruption. Shown is one representative experiment of two independent experiments +/- SD. Statistcal differences were calculated unpaired t-tests. Statistical significance = *$p<0.05$; **$p<0.01$; ***$p<0.001$; ****$p<0.0001$). PHKs = primary human keratinocytes; AHR = aryl hydrocarbon receptor; I3P = indole-3-propionate; I3A = indole-3-aldehyde; L-K = L-kynurenine; MOI = multiplicity of infection; CFU = colony forming units; SD = standard deviation; MVs = membrane vesicles; SE = S. epidermidis; SL = S. lugdunensis; SA = S. aureus

Supplementary Tabelle 1: Primer Sequences

Gene name	Sequence
actin-fw	ttgttacaggaagtcccttgcc
actin-rv	atgctatcacctcccctgtgtg
CYP1A1-fw	gattgagcactgtcaggagaagc
CYP1A1-rv	atgaggctccaggagatagcag
IL1RA-fw	atggagggaagatgtgcctgtc
IL1RA-rv	gtcctgctttctgttctcgctc
TNFAIP3-fw	ctcaactggtgtcgagaagtcc
TNFAIP3-rv	ttccttgagcgtgctgaacagc
HBD3-fw	ttattgcagagtcagaggcggc
HBD3-rv	ctttcttcggcagcattttcggc
RNASE7-fw	gaagaccaagcgcaaagc
RNASE7-rv	cagcagaagcagcagaagg
FLG-FW	acttcactgagtttcttctgatggtatt
FLG-RV	tccagacttgagggtctttttctg
Involucrin-fw	ggtccaagacattcaaccagcc
Involucrin-rv	tctggacactgcgggtggttat
OVOL1-fw	cctcaagagacacgtccgaact
OVOL1-rv	gcgtacttctgctgcacaccat
TSLP-fw	tagcaatcggccacattgcct
TSLP-rv	gaagcgacgccacaatccttg
IL33-fw	gcctgtcaacagcagtctactg
IL33-rv	tgtgcttagagaagcaagatactc
AHR-fw	gtcgtctaaggtgtctgctgga
AHR-rv	cgcaaacaaagccaactgaggtg
Nrf2-fw	cacatccagtcagaaaccagtgg
Nrf2-rv	ggaatgtctgcgccaaaagctg
HMOX1-fw	ccaggcagagaatgctgagttc
HMOX1-rv	aagactgggctctccttgttgc

Supplementary Table 2: Genes included in the RT Profiler Array – Antimicrobial Response

Gene name
CD14
LY96
TLR1
TLR2
TLR4
TLR5
TLR6
TLR9
FADD
IRAK1
IRAK3
IRF5
IRF7
MAP3K7
MYD88
TIRAP
TOLLIP
TRAF6
RIPK1
TICAM1
TICAM2
AKT1
CASP8
LBP
PIK3CA
RAC1
NAIP
NLRC4
NLRP1
NLRP3
NOD1
NOD2
CASP1
PYCARD
BIRC3
CARD6
CARD9
HSP90AA1
MEFV
PSTPIP1
RIPK2
SUGT1
TNF
XIAP
APCS
CRP
DMBT1
ZBP1
CHUK
IKBKB
NFKB1
NFKBIA
RELA
TNFRSF1A
JUN
MAP2K1
MAPK1
MAPK3
MAP2K3
MAP2K4
MAPK14
MAPK8
IFNB1
IL12A
IL12B
IL1B
IL6
MPO
CCL3
CCL5
CXCL1
CXCL2
CXCL8
LYZ
SLC11A1
IFNA1
IL18
BPI
CAMP
CTSG
LCN2
LTF
PRTN3
SLPI
